# Revisional One Anastomosis Gastric Bypass with a 150-cm Biliopancreatic Limb After Failure of Adjustable Gastric Banding: Mid-Term Outcomes and Comparison Between One- and Two-Stage Approaches

**DOI:** 10.1007/s11695-021-05728-9

**Published:** 2021-10-05

**Authors:** Niccolò Petrucciani, Francesco Martini, Marine Benois, Radwan Kassir, Hubert Boudrie, Olivier Van Haverbeke, Celine Hamid, Gildas Juglard, Gianluca Costa, Tarek Debs, Arnaud Liagre

**Affiliations:** 1 Department of Medical and Surgical Sciences and Translational Medicine, Faculty of Medicine and Psychology, St Andrea Hospital, Sapienza University, via di Grottarossa 1035-9, 00189 Rome, Italy; 2 Bariatric Surgery Unit, Clinique des Cedres, Ramsay Générale de Santé, Cornebarrieu, France; 3 Department of Digestive Surgery, CHU Félix Guyon, Saint Denis, La Réunion, France; 4 Division of General Surgery, Campus Bio-Medico Hospital, Rome, Italy; 5 Division of Digestive Surgery and Liver Transplantation, Archet II Hospital, University of Nice-Sophia-Antipolis, Nice, France

**Keywords:** Bariatric surgery, One anastomosis gastric bypass, Adjustable gastric banding, Complications, Revisional surgery

## Abstract

**Purpose:**

Laparoscopic adjustable gastric banding (LAGB) was a common procedure worldwide but associated with a high rate of long-term failure. This study aims to evaluate the safety and effectiveness of conversion to one anastomosis gastric bypass (OAGB) after failed LAGB.

**Materials and Methods:**

We undertook a retrospective analysis of a prospectively maintained database in a tertiary referral center for bariatric surgery. All cases of revisional OAGB with a biliopancreatic limb (BPL) of 150 cm after failed LAGB performed between 2010 and 2016 were analyzed.

**Results:**

Overall, 215 patients underwent conversion from LAGB to OAGB. Indication for surgery was primary weight loss (WL) failure in 30.7% of cases and long-term complications in the remaining patients, with or without associated weight regain. At the time of OAGB, the mean age was 43.2 ± 10.5 years and the mean BMI was 42 ± 6.9. Overall postoperative morbidity was 13.5%. The postoperative abscess ± leak rate was 5.9% in the overall population. Two years after OAGB, 9.7% of patients were lost to follow-up, % excess weight loss (EWL) was 88.2 ± 23.9, and % total weight loss (TWL) was 38.7 ± 9.3. At 5 years, 16.6% of patients were lost to follow-up, %EWL was 82.4 ± 25, and %TWL was 36.1 ± 10. There was no statistical difference in complication rates or WL results between the one-stage and two-stage approaches.

**Conclusion:**

OAGB with a 150-cm BPL represents a safe and effective option after failed LAGB. Both synchronous OAGB and two-step revisional OAGB guarantee satisfying results in terms of postoperative morbidity and WL outcomes.

**Graphical abstract (PLEASE CORRECT THE GRAPHICAL ABSTRACT !!! 215 PATIENTS INSTEAD OF 250:**

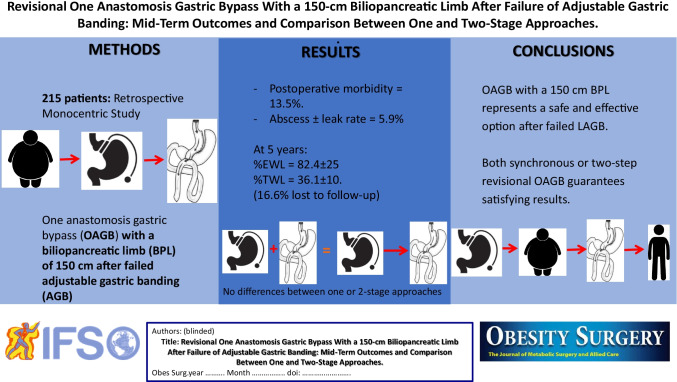

**Supplementary Information:**

The online version contains supplementary material available at 10.1007/s11695-021-05728-9.

## Introduction

Obesity still represents a global health concern. Bariatric surgery is the most effective therapy for morbid obesity, resulting in sustainable weight loss (WL) and an improvement in obesity-related comorbidities [1, 2]. Laparoscopic adjustable gastric banding (LAGB) was the first minimally invasive bariatric procedure to be widely adopted [3]. In the early 2000s in France, LAGB was the technique of choice in 80% of patients undergoing bariatric surgery [3–5]. LAGB has been widely practiced in France since 1995, with more than 160,000 procedures performed to date [6]. On a worldwide scale, the number of LAGB procedures represented 24.4% of the total bariatric procedures in 2003, although it decreased to 5% in the last International Federation for the Surgery of Obesity and Metabolic Disorders (IFSO) report in 2018 [7, 8].

Even though satisfactory WL results have been reported after LAGB placement and some evidence still supports the choice of a gastric band in selected patients (9), the majority of authors have abandoned LAGB because of high early failure rates and/or inferiority compared to other bariatric procedures [9–11]. Long-term complications include band erosion, band migration, pouch dilatation, intractable nausea, reflux, and port infection [12]. As a result, the adjustable gastric banding (AGB) removal rate has increased over time (about 3–4% per year), and since 2012, more bands have been removed than have been placed. At present, almost half of the placed bands have been removed [6, 11, 13].

In most cases, patients who have their AGB removed regain weight to presurgery levels. Aarts et al. [14] reported a complete regain of weight at 5 years after LAGB removal in all 21 patients of their series. Rohner et al. [15] reported similar bariatric results in 21 patients at 5 years. Moreover, they found that removal of the banding system alone leads to the deterioration of physical and psychiatric comorbidities, as well as low quality of life scores.

Therefore, a second bariatric procedure should be proposed to all patients when deemed technically feasible and safe. Unfortunately, revisional procedures carry a higher complication rate than their primary counterparts; in particular, the risk of a staple line leak is significantly greater [16].

There is no consensus about which conversional procedure should be offered. The comparison concerns the safety and efficacy of the different procedures. The most common revisional procedures after LAGB failure are the Roux-en-Y gastric bypass (RYGB) and the sleeve gastrectomy (SG) [17]. One anastomosis gastric bypass (OAGB) has been recently proposed by several teams with promising results[18–20]. AGB withdrawal and revisional surgery may be performed in one or two stages. The choice between the two methods is still a matter of debate, such as the optimal time lapse between the two procedures in the case of a two-stage approach.

This study aims to evaluate the safety and effectiveness of conversion to OAGB after failed LAGB in a referral institution for bariatric surgery.

## Patients and Methods

Between May 2010 and December 2016, 215 patients underwent OAGB as a revisional procedure after failed LAGB. Patients were retrieved from a prospectively maintained database of all bariatric procedures performed in our institution. The institutional review board approved the present study.

### Preoperative Workup

Failure of LAGB was defined as insufficient WL (excess weight loss (EWL) > 50% at 18-month follow-up [21]) and/or development of long-term complications, including band slippage/displacement, and pouch dilatation. Preoperative workup included upper gastrointestinal (GI) endoscopy, upper GI series, abdominal ultrasound, and clinical, biochemical, nutritional, and psychological assessments. The multidisciplinary obesity board of the institution validated the indication for revisional surgery.

The patients were classified into three groups according to the timing of conversion to OAGB. Patients in the first group (group 1) underwent LAGB removal and synchronous OAGB. In group 2, conversion to OAGB was performed within 12 months from LAGB removal. In group 3, OAGB was performed 12 months or more after LAGB removal.

### Surgical Technique

All revisional procedures after LAGB were performed by an experienced bariatric surgeon with a standardized procedure. OAGB was performed concomitantly with lap-band removal when possible (1-stage procedure) or after a delay (2-stage procedure). The band was always deflated a few weeks before the surgical procedure. At first, the port was liberated and exteriorized from the skin, then the band was identified and dissected from its attachments to the liver; the gastrogastric valve was taken down carefully, and the angle of His was identified. The fibrous capsule surrounding the band was dissected at the level of the His angle to liberate the left crus, and the rest of the scar tissue was not removed.

At this time, the operating surgeon estimated if local conditions allowed a 1-stage procedure or not.

The gastric bypass was performed as previously reported [2]. The lesser sac was entered at the crow’s foot, and a long and narrow gastric pouch was fashioned over a 36-Fr calibration tube. The omentum was divided in patients with central obesity to facilitate the ascent of the jejunum. A 150-cm jejunal loop was measured from the Treitz ligament using marked graspers and then an antecolic side-to-side gastrojejunostomy was fashioned using a 60-mm linear stapler.

### Postoperative Outcomes and Follow-up

In the postoperative period, oral liquid intake was resumed at postoperative day 1, and if liquid intake was tolerated, solid intake was subsequently resumed. Postoperative complications were classified according to the Clavien–Dindo classification [22].

Follow-up was performed at 1 month, 3 months, and 6 months and then every 6 months thereafter and consisted of physical examination and blood tests. The percentage of EWL (%EWL) was calculated using the maximum weight as the initial weight. The %EWL was calculated as [initial weight – follow-up (FU) weight] / [initial weight − ideal weight] × 100. The ideal weight was set as that equivalent to a BMI of 25 kg/m^2^. The percentage of total weight loss (%TWL) was calculated using the following formula: (weight loss / initial weight) × 100. Residual %TWL and %EWL were defined as the WL obtained with the primary treatment (LAGB) at the time of secondary treatment (OAGB). Additional %TWL after OAGB was defined as (weight loss/initial weight) × 100, using the weight as the time of OAGB as initial weight.

The evolution of obesity-related comorbid conditions was assessed according to the use and discontinuation of medication postoperatively in the instance of diabetes, hypertension, and dyslipidemia. Remission of hypertension was defined as a systolic blood pressure of less than 130 mmHg or a diastolic blood pressure of less than 85 mmHg without the use of antihypertensive drugs. Improvement was defined as a decrease in the quantity or dosage of antihypertensive drugs. Diabetes remission was defined as fasting glucose of less than 5.6 mmol/L and a glycosylated hemoglobin value of less than 6.5% without the use of oral hypoglycemic medications or insulin. Improvement was defined as a decrease in the quantity or dosage of oral hypoglycemic medications or insulin. The presence of preoperative sleep apnea syndrome was quantified by sleep studies and postoperative resolution by discontinued use of continuous positive airway pressure masks.

Biliary reflux was defined as the presence of clinical symptoms necessitating treatment, such as heartburn and/or bile vomiting and/or biliary regurgitation, particularly during the night or in dorsal decubitus. Some patients reported mild episodes of hypoglycemia, defined as episodic feelings of faintness between meals (to differentiate from dumping syndrome), associated with glucose values of at least lower than 70 mg/dL at a glucometer self-assessment.

### Statistical Analysis

Data were expressed as mean ± standard deviation or median (range) or as numbers and percentages. Comparisons were made using the chi-square test for nominal data and Student’s *t* test for continuous data. A *p* value < 0.05 was considered statistically significant. All statistical analyses were performed using SPSS software, version 25.

## Results

### Patients’ Characteristics

**Fig. 1:**
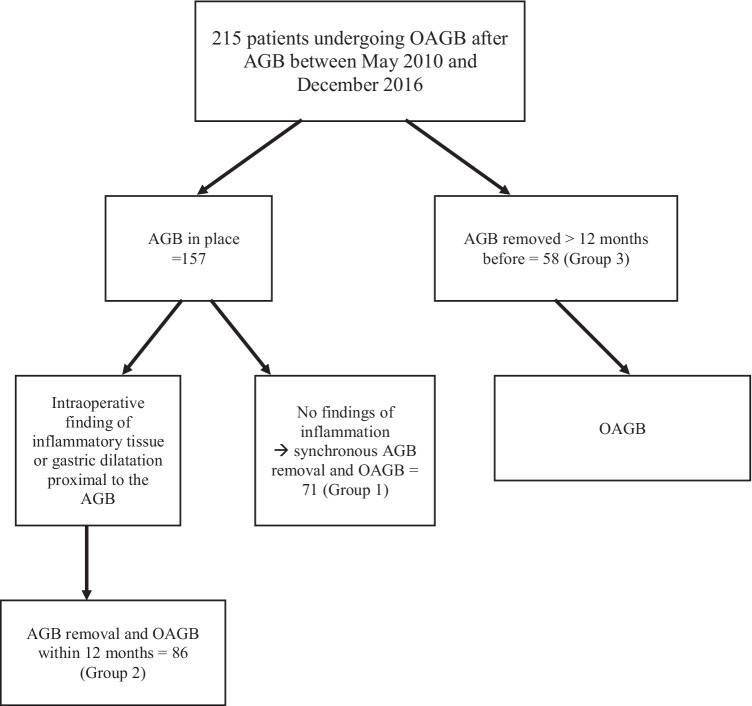
Flow chart of the patients included

During the study period, 215 patients underwent conversion from LAGB to OAGB. A flow chart and the timing between AGB removal and OAGB in patients are reported in Fig. 1. Indication for surgery was primary WL failure in 30.7% of cases and long-term complications from the AGB (band slippage/displacement, pouch dilatation) in the remaining patients, with or without associated weight regain.

Out of the 215 patients included, 195 were females. Before LAGB, the mean weight was 119.7 ± 19.3, with a BMI of 44.4 ± 6.4. Minimal weight and BMI after LAGB were 85 ± 18.6 and 31.8 ± 6.5, respectively, with a maximal %EWL of 66.9 ± 29 and a maximal %TWL of 28.6 ± 12.1. The mean timeframe between AGB removal and OAGB was 14.1 ± 25.5 months, and the mean period between LAGB surgery and OAGB was 102.9 ± 42 months.

At the time of OAGB, the mean age was 43.2 ± 10.5 years (range: 23–68); the mean weight was 113 ± 21.6, with a mean BMI of 42 ± 6.9. The characteristics of patients are reported in Table 1.

**Table 1:** Characteristics of the included patients

*N*	215
Age at the time of OAGB	43.2 ± 10.5 (23–68)
Sex: females, *n* (%)	195 (90.7)
Weight before LAGB (kg)	119.7 ± 19.3 (66–233)
BMI before LAGB (kg/m^2^)	44.4 ± 6.4 (33–75)
Maximal %EWL after LAGB	66.9 ± 29 (0–165)
Maximal %TWL after LAGB	28.6 ± 12.1 (0–60.7)
Minimal weight with AGB (kg)	85 ± 18.6 (44–150)
Minimal BMI with AGB (kg/m^2^)	31.8 ± 6.5 (16.3–56.2)
Maximal efficacy of AGB in terms of weight loss (%EWL > 50), *n* (%)	150 (69.8)
Time between LAGB and OAGB (months)	102.9 ± 42 (0–133)
Time between AGB removal and OAGB (months)	14.1 ± 25.5 (22–200)
Reasons for AGB removal, *n* (%)	
Intolerance to AGB	15 (6.9)
Proximal gastric dilatation and weight regain	115 (53.4)
Perforation	10 (4.6)
Insufficient weight loss or weight regain	68 (31.6)
Small bowel obstruction	1 (0.04)
Others	4 (1.8)
Age at the time of OAGB	43.2 ± 10.5 (23–68)
Weight before OAGB (kg)	113 ± 21.6 (66–233)
BMI before OAGB (kg/m^2^)	42 ± 6.9 (27–67)
Residual %EWL	10.5 ± 29.6 (− 124–83)
Residual %TWL	5.4 ± 11.8 (− 30.1–39.2)
Patients with higher weight at OAGB than at LAGB, *n* (%)	63 (29.3)
Efficacy of AGB in terms of weight loss before OAGB (%EWL > 50), *n* (%)	16 (7.4)

Data are presented as mean ± SD and range for continuous variables, as the absolute number and percentages for categorical variables

### Postoperative Short and Long-Term Complications

All the OAGBs were performed with a laparoscopic approach. No postoperative mortality occurred. Postoperative short-term and long-term complications are listed in Tables 2 and 3. Overall postoperative morbidity was 13.5%. The postoperative abscess ± leak rate was 5.9% in the overall population. All the leaks occurred at the staple line in the area where the band had been placed. Among long-term complications, internal hernias occurred in 3.7% of patients. Gastro-esophageal reflux that was resistant to medical treatment occurred in 21.3% of patients and required conversion to RYGB in 4.2% of cases. Mild episodes of hypoglycemia occurred in 1% of patients, episodes of diarrhea in 0.5%, and anastomotic ulcers in 0.5%.

**Table 2:** Postoperative early complications after revisional OAGB for failed LAGB

Short-term complications	*N* (%)	Clavien–Dindo grading
Death	0 (0)	
Perianastomotic abscess ± leak	12 (5.6)	Grade 2 (*n* = 1)
		Grade 3a (*n* = 6)
		Grade 3b (*n* = 4)
		Grade 4 (*n* = 1)
Pneumonia/atelectasis	5 (2.3)	Grade 2 (*n* = 5)
Small bowel perforation	1 (0.5)	Grade 3b (*n* = 1)
Anastomotic inflammation without abscess	1 (0.5)	Grade 2 (*n* = 1)
Phlebitis	1 (0.5)	Grade 2 (*n* = 1)
Myocardial infarction	1 (0.5)	Grade 2 (*n* = 1)
Pneumothorax	1 (0.5)	Grade 3a (*n* = 1)
GI bleeding without the need of transfusion	1 (0.5)	Grade 1 (*n* = 1)
Bleeding from the drain without the need of transfusion	4 (1.9)	Grade 1 (*n* = 4)
Anastomotic stenosis	2 (0.9)	Grade 3a (*n* = 2)
Total	29 (13.5)

**Table 3:** Postoperative late complications and additional procedures after revisional OAGB for failed LAGB

Complication/additional procedure	*N* (%)
Reflux	46 (21.4)
Medical treatment	37 (17.2)
Surgical treatment	9 (4.2)
Internal hernia	8 (3.7)
Anastomotic ulcer	4 (1.9)
Medical treatment	3 (1.4)
Surgical treatment	1 (0.5)
Insufficient weight loss	1 (0.5)
Treated with banding of the gastroplasty	
Invalidating diarrhea	13 (6.0)
Medical treatment	12 (5.6)
Surgical treatment	1 (0.5)
Hypoglycemia	2 (0.9)
Medical treatment	2 (0.9)
Surgical treatment	0 (0)
Intestinal invagination	1 (0.5)
Incisional hernia	4 (1.9)
Suicide	1 (0.5)
Cholecystectomy	17 (7.9)
Laparoscopic exploration for abdominal pain	2 (0.9)

One patient died during follow-up from suicide. Compliance with vitamin supplementation was declared by 81.8% of patients. Iron infusions were administered to 6.5% of patients during follow-up. No cases of malnutrition requiring hospitalization or OAGB reversal were observed. Results of blood tests are reported in Table 4.

**Table 4:** Results of blood tests of patients who underwent OAGB as a revisional procedure after failed LAGB

Variable	Preoperative, % abnormal	12 months, % abnormal	24 months, % abnormal
Hemoglobin (g/dL)	13.5 ± 1.1 (*N* = 205), 5.8%	13.3 ± 1.2 (*N* = 67), 7.5%	13.2 ± 1.3 (*N* = 47), 9%
Albumin (g/dL)	38.3 ± 3.7 (*N* = 144), 14.5%	40.9 ± 3.7 (*N* = 63), 7%	39.9% ± 3.6 (*N* = 42), 5%
Ferritin (μg/L)	145 ± 126 (*N* = 145), 6.2%	84.9 ± 80.9 (*N* = 64), 9.4%	71.6 ± 56.1 (*N* = 42), 4.8%
Prealbumin (g/L)	0.24 ± 0.05 (*N* = 108), 14.8%	0.22 ± 0.05 (*N* = 37), 24.4%	0.23 ± 0.05 (*N* = 32), 11.9%
Vitamin A (μmol/L)	2.23 ± 0.7 (*N* = 91), 17.5%	1.46 ± 0.62 (*N* = 59), 88%	1.75 ± 0.59 (*N* = 37), 62.2%
Vitamin B_1_ (nmol/L)	NR	148.3 ± 33.8 (*N* = 22), 0%	153.4 ± 44.5 (*N* = 7), 14.3%
Vitamin B_9_ (ng/L)	16 ± 5.5 (*N* = 141), 2.8%	20.3 ± 14.5 (*N* = 63), 19.1%	21.2 ± 14.2 (*N* = 40), 22.5%
Vitamin B_12_ (pmol/L)	319 ± 107 (*N* = 145), 2%	313 ± 140.3 (*N* = 60), 3.4%	322 ± 146.6 (*N* = 43), 0%
Vitamin D (ng/mL)	42 ± 20 (*N* = 144), 87.5%	69.5 ± 27.2 (*N* = 65), 60%	70.5 ± 26.4 (*N* = 43), 58.2%
Parathyroid hormone (pg/mL)	NR	61.4 ± 29.2 (*N* = 33), 60.4%	56.1 ± 24.7 (*N* = 31), 61.3%
Calcium (mmol/L)	2.35 ± 0.1 (*N* = 151), 0.6%	2.33 ± 0.1 (*N* = 66), 3%	2.29 ± 0.09 (*N* = 44), 4.6%

### Weight Loss Outcomes (Table 5) and Resolution of Comorbidities

**Table 5:** Weight loss results of patients who underwent revisional OAGB after LAGB

Variable	Mean ± SD or *N* (%)
*At 12-month follow-up after OAGB (n* = *215)*	
Weight	80.4 ± 17.1 (50–168)
BMI	29.8 ± 5.6 (18–53)
Lost to follow-up	15 (6.9%)
%EWL	78.8 ± 21.7 (19–143)
%TWL	34.3 ± 8.8 (9–56.6)
Additional %TWL	28.4 ± 8.9 (6.2–50.5)
*At 24-month follow-up after OAGB (n* = *215)*	
Weight	75.2 ± 17.3 (48–175)
BMI	28 ± 5.5 (17–51)
Lost to follow-up	21 (9.7%)
%EWL	88.2 ± 23.9 (28–158)
%TWL	38.7 ± 9.3 (10–62.9)
Additional %TWL	33.2 ± 9.7 (6.2–58.4)
*At 60-month follow-up after OAGB (n* = *168)*	
Weight	78.2 ± 16.9 (51–130)
BMI	29.2 ± 5.8 (18–52)
Lost to follow-up	28 (16.6%)
%EWL	82.4 ± 25 (29–158)
%TWL	36.1 ± 10 (12.9–61.1)
Additional %TWL	30.5 ± 11 (− 4.3–52.35)
*At* > *84-month follow-up after OAGB (n* = *110)*	
Weight	79.1 ± 17.8 (51–130)
BMI	29.7 ± 6.4 (18–54)
Lost to follow-up	31 (28.1%)
%EWL	80.2 ± 28.3 (28–158)
%TWL	33.9 ± 10.2 (9.5–56.2)
Additional %TWL	27.7 ± 10.9 (− 2.1 to 52.5)

Data are presented as mean ± standard deviation (range) or as number (percentage)

At 2 years after OAGB, 9.7% of patients were lost to follow-up, BMI was 28 ± 5.5 kg/m^2^, %EWL was 88.2 ± 23.9, and %TWL was 38.7 ± 9.3. At 5 years after OAGB, 16.6% of patients were lost to follow-up, BMI was 29.2 ± 5.8 kg/m^2^, %EWL was 82.4 ± 25, and %TWL was 36.1 ± 10. Table 5 reports WL outcomes. Table 6 reports comorbidities and their evolution.

**Table 6:** Evolution of comorbidities after revisional OAGB

Comorbidity	Before OAGB	At last follow-up	Regression (%)
Arterial hypertension	14.4% (31/215)	Lost to follow-up = 2	72
		Resolution = 21	
Diabetes	7% (15/215)	Lost to follow-up = 5	90
		Resolution = 9	
OSAS	4.1% (9/215)	Lost to follow-up = 0	77
		Resolution = 7	
Dyslipidemia	7% (15/215)	Lost to follow-up = 1	100
		Resolution = 14	

### Comparison of AGB Efficacy in Terms of Weight Loss

Table 7 (supplementary) reports patients’ characteristics and outcomes according to the efficacy of the LAGB (defined as %EWL > 50 at 18-month follow-up).

### Timing of OAGB After AGB Removal

Table 7 reports patients’ characteristics and outcomes according to the timing of OAGB after AGB removal, comparing synchronous OAGB (group 1) versus delayed OAGB within 12 months (group 2), versus delayed OAGB > 12 months (group 3). Weight and BMI were lower in group 1. The three groups had similar rates of postoperative complications and comparable WL results at 60-month follow-up. Table 9 (supplementary) reports the rates of postoperative leak and conversion to RYGB because of reflux that was resistant to medical treatment, according to the reason and timing of AGB removal and OAGB fashioning.

**Table 7:** Patients’ characteristics and outcomes according to the timing of OAGB after AGB removal

	Synchronous OAGB	OAGB within 12 months	OAGB > 12 months	*p*
	Group 1	Group 2	Group 3	
	*N* = 70	*N* = 87	*N* = 58	
Age at the time of OAGB	42.5 ± 10.5	44.8 ± 10.2	41.9 ± 10.9	0.201
Weight before AGB	116.9 ± 17.8	124.1 ± 21.1	116.7 ± 17.4	**0.025**
BMI before AGB	43.3 ± 5.7	45.7 ± 7.3	43.9 ± 5.8	**0.060**
Minimal weight with AGB	82.5 ± 18.9	87.2 ± 19.9	85.4 ± 16.1	0.292
Minimal BMI with AGB	30.8 ± 6.5	32.3 ± 7.0	32.3 ± 5.7	0.294
%TWL maximum with AGB	29.3 ± 12.3	29.4 ± 12.7	26.5 ± 11.1	0.295
%EWL maximum with AGB	70.2 ± 31.4	66.8 ± 28.2	63.2 ± 27.2	0.402
Raison for AGB removal				
Intolerance	1 (1.4%)	2 (2.3%)	12 (20.8%)	** < 0.001**
Proximal gastric dilatation	36 (51.4%)	46 (53%)	34 (58.6%)	
Perforation	0	5 (5.7%)	5 (8.6%)	
Insufficient weight loss	31 (44.3%)	33 (37.9%)	5 (8.6%)	
Bowel obstruction	0	1 (1.1%)	0	
Other	2 (2.9%)	0	2 (3.4%)	
Reason of not doing AGB removal and OAGB in the same time				
Proximal gastric dilatation		45 (51.7%)		** < 0.001**
Preoperative findings of inflammation		32 (36.8%)		
Endoscopic AGB removal for intragastric AGB perforation		2 (2.3%)		
Preoperative findings of catheter obstruction		1 (1.1%)		
Preoperative findings of liver steatosis		1 (1.1%)		
Other		6 (7.0%)		
Time between LAGB and OAGB	92.8 ± 40.7	107.3 ± 42.4	108.7 ± 41.5	** < 0.001**
Months between AGB removal and OAGB	–	4.4 ± 2.3	45.7 ± 32.2	** < 0.001**
GERD	32 (45.7%)	31 (35.6%)	16 (27.6%)	0.102
Residual %TWL	7.8 ± 11.1	6.3 ± 1.2	1.2 ± 12.6	**0.004**
Weight before OAGB	102.5 ± 20.6	116.3 ± 23.6	116.7 ± 17.4	**0.019**
BMI before OAGB	39.8 ± 6.5	42.8 ± 7.3	43.7 ± 6.5	**0.003**
Postoperative leak after OAGB	4 (5.7%)	7 (8%)	1 (1.7%)	0.267
Overall morbidity after OAGB	9 (12.9%)	13 (14.9%)	5 (8.6%)	0.529
BMI 24 months after OAGB	27.1 ± 4.4	29.4 ± 6.2	27.0 ± 5.6	**0.014**
%EWL 24 months after OAGB	91.5 ± 23.9	82.5 ± 22.7	92.8 ± 24.5	**0.022**
%TWL 24 months after OAGB	38.4 ± 8.7	37.2 ± 9.4	41.7 ± 9.4	**0.030**
Additional %TWL 24 months	31.0 ± 9.0	31.7 ± 9.5	38 ± 9.1	** < 0.001**
BMI 60 months after OAGB	28.1 ± 4.9	30.2 ± 5.9	29.1 ± 6.6	0.163
%EWL 60 months after OAGB	86.4 ± 25.8	78.5 ± 23.9	84.2 ± 25.6	0.245
%TWL 60 months after OAGB	36.2 ± 9.2	35.4 ± 10.6	35.4 ± 10.6	0.669
Additional %TWL 60 months	28.9 ± 10.5	29.2 ± 11.3	34.5 ± 10.2	**0.042**
Conversion to RYGB for invalidating reflux	1 (1.4%)	6 (6.9%)	2 (3.4%)	0.256

Preoperative and postoperative variables are compared between patients undergoing synchronous AGB removal and OAGB and delayed OAGB after AGB removal. Data are presented as mean ± SD for continuous variables, as absolute number and percentages for categorical variables (one-way ANOVA). Significant values are reported in bold

## Discussion

This report presents the mid-term outcomes of a cohort of 215 patients who underwent OAGB with a 150-cm BPL as a revisional procedure after failed LAGB. The results suggest that this procedure guarantees satisfying outcomes in terms of postoperative morbidity and WL. Synchronous and two-step revisional OAGB provide comparable results.

LAGB in the past represented a very common bariatric procedure worldwide, because of its technical simplicity and short-term efficacy [4, 23]. However, recently it has become apparent that LAGB is associated with a remarkable rate of long-term complications, and that AGB removal is frequent, with a rate as high as 40% at 7-year follow-up [13]. Therefore, revisional surgery after failed LAGB has become common [24, 25].

Two main questions arise for surgeons treating patients with LAGB failure, in whom revisional surgery has been decided after multidisciplinary evaluation. The first question concerns which operation should be recommended. Several options are possible, the most frequent being RYGB and SG [26]. The second question is the timing between AGB removal and the revisional procedure. The surgeries may be performed in one step or during two different operations with a variable delay [27, 28].

OAGB is a more recent bariatric procedure, which has rapidly gained acceptance and diffusion worldwide, and represents 7.6% of all bariatric operations [7, 29]. It has been recognized by the IFSO as a mainstream bariatric procedure [30], and several studies including thousands of patients have established the efficacy and safety of this procedure in treating obesity and its related comorbidities [31–33]. OAGB has been demonstrated to be effective in the setting of revisional bariatric surgery [34, 35].

The present study demonstrates that OAGB is a safe and effective option after the failure of LAGB. In our series of 215 patients, we had a very low rate of severe morbidity and no postoperative deaths. Long-term malnutrition was not observed, and the long-term complication rate was low, the most frequent being internal hernia with a rate of 3.7% and reflux that is resistant to medical treatment (4.2%) [36]. Internal hernia after OAGB has been reported as a potential complication, even if it is associated with a low rate of bowel ischemia and a need for intestinal resection [37].

Weight loss outcomes were very encouraging, with a %EWL of 88.2 ± 23.9 and a %TWL of 38.7 ± 9.3 at 2-year follow-up. At 5-year follow-up, BMI was 29.2 ± 5.8 kg/m^2^, %EWL was 82.4 ± 25, and %TWL was 36.1 ± 10. We did not find more overall complications or worst results between patients having synchronous AGB removal and OAGB and those having two-step revisional surgery, within 12 months or after 12 months. Even the variation of timing at the second step, within 1 year or after at least 1 year, did not cause statistically significant variations in the postoperative outcomes in our series. However, we emphasize that the lower rate of postoperative leaks was reported in the group of delayed OAGB > 12 months, and it was as low as 1.7%, versus 8% in patients with delayed OAGB at < 1 year and 5.7% in the synchronous procedure. These results were not statistically significant, but this may be related to the number of the included patients. Among the long-term complications, reflux that is resistant to medical treatment is possible and is usually treated with conversion to RYGB; even for this complication, no significant differences were found between the three groups.

The decision to convert the OAGB for reflux resistant to medical treatment is based not only on esophageal impedance PH testing [38], but on a complete assessment also including computed tomography with oral contrast ingestion and upper gastrointestinal endoscopy. In our experience, conversion to RYGB was effective in > 90% of patients [36]. Hiatal hernia should be systematically searched for as it may be responsible for the reflux, and in these cases, surgical treatment of the hiatal hernia may permit the remission of the reflux.

In our experience, OAGB after AGB is associated with a higher rate of leaks and transformation to RYGB for reflux, compared to OAGB as a primary procedure [32]. Even if no significant differences were found, a trend for lower rates of leaks was found in patients with OAGB fashioned more than 12 months after AGB removal, whereas similar leak rates were found in patients with synchronous procedures or AGB removal and OAGB within 12 months.

Our results show that OAGB as a revisional procedure after LAGB has a very satisfying profile in terms of safety and efficacy. The advantages of OAGB as a revisional procedure after LAGB are that the scar tissue due to the band does not need to be completely removed because the anastomosis is performed much lower. The dissection is done through the omental bursa, and the preparation of the gastrojejunal anastomosis is done in fresh and “healthy tissue.” It should be noted, as reported previously, that our OAGB technique consists in the fashioning of the anastomosis at 150 cm from the Treitz ligament [39]. Strengths of the present study are the large number of included patients (to our knowledge, it represents the largest single-institution series), the standardized surgical technique, and the remarkable 5-year follow-up rate.

Several authors have studied the outcomes of revisional procedures after failed LAGB. The first studies reported conversion to RYGB [40] or compared the outcomes of revisional SG versus RYGB, with no significant differences in postoperative outcomes [26, 41].

Al-Kurd et al. [42] compared RYGB after failed LAGB versus primary RYGB, including 161 patients in both groups. They showed no differences in short-term and long-term postoperative morbidity rates (7.5% in the revisional group versus 11.8% in the primary RYGB, non-significant) but better WL results for primary RYGB (61.5% vs. 75.5% of EWL, respectively, with 6-month follow-up attained in 78% of the patients).

Poublon et al. [43] recently published an interesting and well-conducted study, comparing OAGB versus RYGB after the failure of LAGB or SG. They included 306 revisional RYGB and 185 revisional OAGB. Previous bariatric surgery consisted of SG in 28.5% of patients and LAGB in the remaining cases. Intra-abdominal complications (leakage, bleeding, intra-abdominal abscess, and perforation) were significantly less frequent after revisional OAGB (1.1% vs. 4.9%, *p* = 0.025). However, revisional surgery for biliary reflux (5.4% vs. 0.3%, *p* < 0.001) was more prevalent in the OAGB group. On the other hand, surgical intervention for internal herniation (0.0% vs. 4.9%, *p* = 0.002) was more common in the RYGB group. Concerning WL results, OAGB guaranteed better outcomes, with larger %TWL at 12 months (mean 24.1 ± 9.8 vs. 21.9 ± 9.7, *p* = 0.023) and 24 months (mean 23.9 ± 11.7 vs. 20.5 ± 11.2, *p* = 0.023) of follow-up. A greater % excess BMI loss (EBMIL) was also reported for OAGB [43].

Chansaenroj et al. [44] published concordant results. The authors included 53 patients undergoing OAGB [24], SG [16], and RYGB [45] after failed LAGB. In this study, patients who underwent revisional OAGB had better WL at 1- and 2-year follow-ups. However, no significant differences in %EWL were reported. Similarly, in a series by Almalki et al. [46] that included 116 patients who underwent OAGB (81) or RYGB (35) after a failed restrictive bariatric procedure, OAGB was associated with better WL results. At 1-year follow-up, %EWL was 76.8% in the OAGB group versus 32.9% in the RYGB group. The major morbidity rate was 10% in the overall population without significant differences between the two groups.

Data about the long-term results of conversion from LAGB to OAGB are lacking. Only Bruzzi et al. [47] report data of 30 patients who underwent OAGB after failed restrictive procedures. In this series, the major complication rate was 10% and two patients required conversion from revisional OAGB to RYGB for resistant reflux. At 5 years, the %EBMIL was 66%. The results of this series are satisfying and comparable with the long-term results of our present series, showing the long-term efficacy of revisional OAGB.

Parmar et al. [48] systematically reviewed the literature retrieving 17 studies including 1075 cases of OAGB after failed LAGB, SG, vertical banded gastroplasty, and gastric plication. Patients had a median limb length of 200 cm, which differs from our technique [39]. The leak rate of this series was 1.54%, and the marginal ulcer rate was 2.44%. Mortality was 0.3%, and the %EWL at 1 year and 2 years was 65.2% and 68.5%, respectively. The mean follow-up was 2.44 years.

AGB removal and conversional surgery may be performed concomitantly or in a staged fashion. The more appropriate approach remains a topic of discussion. Surgeons in favor of a single stage argue that this method requires fewer total surgeries and avoids weight regain, which constantly follows a period of non-restriction [49]. Those in favor of a two-stage approach claim that the interval between procedures allows for inflammation to be reduced and for vascularization to be improved at the fibrotic portion of the stomach where the band was placed, therefore limiting the risk of staple line leak [50].

We did not find more overall complications or worst results between patients having synchronous and two-step revisional surgery. Even the variation of timing of the second step, within 1 year or after at least 1 year, did not cause statistically significant major variations in the postoperative outcomes in our series. However, we emphasize that a lower rate of postoperative leaks was reported in the group with the OAGB delayed for > 12 months, and it was as low as 1.7%, versus 8% in patients with OAGB delayed for < 1 year and 5.7% in the synchronous procedure. These results were not statistically significant, but this may be related to the high number of patients included. Among long-term complications, reflux that was resistant to medical treatment is possible and is usually treated with conversion to RYGB; even for this complication, no significant differences were found between the three groups.

In our experience, OAGB after AGB is associated with a higher rate of leaks and transformation to RYGB for reflux, compared to OAGB as a primary procedure. Even if no significant differences were found, a trend for a lower rate of leaks was found in patients with OAGB fashioned more than 12 months after AGB removal, whereas similar rates of leaks were found in patients with synchronous procedures or AGB removal and OAGB within 12 months.

Lessing et al. [51] reported data on 57 patients undergoing synchronous (41 patients) or two-step conversion from LAGB to OAGB. The complication rate was 15.7%, and one postoperative death occurred. The mean %EWL was 64.5% at 1-year follow-up, and no differences were reported between synchronous and two-step cases. Schäfer et al. [28] analyzed the timing of conversion from LAGB to RYGB in a series of 165 patients, reporting a major complication rate of 15.3% for one-stage surgeries versus 16.9% for two-step procedures and no significant differences.

Our results show that OAGB as a revisional procedure after LAGB has a very satisfying profile in terms of safety and efficacy. It should be emphasized that our OAGB technique fashions the anastomosis at 150 cm from the Treitz ligament. Strengths of the present study are the large number of patients included (to our knowledge, it represents the largest single-institution series) and the standardized surgical technique.

### Limits

The present study has several limitations including its single-center and retrospective design. However, the number of patients is remarkable considering the single-center design of the study. The 5-year follow-up rate of more than 80% may be considered adequate in the context of bariatric literature where very few published studies have an FU of 70% or more [52].

## Conclusion

OAGB with a biliopancreatic limb of 150 cm represents a safe and effective option after failed LAGB. Both synchronous OAGB and two-step revisional OAGB guarantee satisfying results in terms of postoperative morbidity and WL outcomes.

## Supplementary Information

Below is the link to the electronic supplementary material.

Supplementary file1 (DOCX 21 KB)
