# Supplementary material for: Revisional One Anastomosis Gastric Bypass with a 150-cm Biliopancreatic Limb After Failure of Adjustable Gastric Banding: Mid-Term Outcomes and Comparison Between One- and Two-Stage Approaches
Source: Obes Surg. 2021 Oct 5;31(12):5330–41. doi: 10.1007/s11695-021-05728-9 (PMC8595146; doi:10.1007/s11695-021-05728-9)
Supplement: Supplementary file 1 — Supplementary file1 (DOCX 21 KB) [file 11695_2021_5728_MOESM1_ESM.docx]

Table 7 Comparison between patients with effective AGB in terms of weight loss and those with insufficient weight loss after LAGB

|  | Effective AGB | Ineffective AGB | *p* |
| --- | --- | --- | --- |
|  | *N* = 150 | *N* = 65 |  |
| Age at the time of OAGB | 43.3 ± 10.5 | 43.2 ± 10.7 | 0.953 |
| Sex: females | 138 (92%) | 59 (90.7%) | 0.319 |
| Reasons for AGB removal | | | |
| Intolerance to AGB | 15 (10%) | 10 (15.3%) | **0.001** |
| Proximal gastric dilatation and weight regain | 89 (59.4%) | 20 (30.7%) |  |
| Perforation | 5 (3.3%) | 5 (7.6%) |  |
| Insufficient weight loss or weight regain | 36 (24.1%) | 30 (46.2%) |  |
| Small bowel obstruction | 1 (0.6%) | 0 (0%) |  |
| Others | 4 (2.6%) | 0 (0%) |  |
| Weight before LAGB | 118.8 ± 19.5 (80–220) | 123 ± 18.4 (92–180) | 0.102 |
| BMI before LAGB | 43.8 ± 6.3 (33–65) | 45.6 ± 6.6 (36–75) | 0.060 |
| Maximal %EWL after LAGB | 81.5 ± 20.7 (50–165) | 33.2 ± 12.4 (0–48) | **< 0.001** |
| Maximal %TWL after LAGB | 34.3 ± 9.2 (9.6–60.7) | 15.4 ± 6.5 (29.6–66) | **< 0.001** |
| Minimal weight with AGB | 77.2 ± 14.2 (44–150) | 103.6 ± 14 (80–142) | **< 0.001** |
| Minimal BMI with AGB | 28.8 ± 4.5 (16.3–43.6) | 38.8 ± 4.8 (29–56.2) | **< 0.001** |
| Weight before OAGB | 108.9 ± 21 (66–233) | 123.5 ± 19.8 (90–186) | **< 0.001** |
| BMI before OAGB | 40.4 ± 6.4 (27–67) | 45.8 ± 6.6 (34–67) | **< 0.001** |
| Patients with weight at the time of OAGB > weight at the time of AGB | 32 (21.3%) | 28 (43%) | **0.001** |
| Residual %EWL | 16.3 ± 28.3 (− 110–83) | − 2.7 ± 28.5 (− 124–49) | **< 0.001** |
| Residual %TWL | 7.7 ± 11.3 (− 22.5–39.2) | 0.09 ± 11.2 (− 30.1–33.3) | **< 0.001** |
| Reflux before OAGB | 56 (37.3%) | 22 (33.8%) | 0.786 |
| Weight at 24 months after OAGB | 72.5 ± 16.4 (48–175) | 81.3 ± 18 (49–140) | **0.001** |
| BMI at 24 months after OAGB | 27 ± 4.9 (17–51) | 30.3 ± 6.3 (21–50) | **< 0.001** |
| %EWL at 24 months after OAGB | 92.1 ± 22.6 (10–63) | 78.5 ± 24.4 (13.6–58.3) | **< 0.001** |
| %TWL at 24 months after OAGB | 39.7 ± 8.6 | 36.4 ± 10.6 | **0.028** |
| Additional %TWL 24 months | 33.2 ± 9.4 (6.2–58.4) | 33.2 ± 10.4 (13.2–53.5) | 0.827 |
| Weight at 60 months after OAGB | 75.3 ± 14.5 (51–128) | 84.3 ± 20 (56–130) | **0.004** |
| BMI at 60 months after OAGB | 28.2 ± 4.8 (18–39) | 31.5 ± 7 (20–52) | **0.001** |
| %EWL at 60 months after OAGB | 86.6 ± 23.7 (30–158) | 73.3 ± 25.6 (29–129) | **0.003** |
| %TWL at 60 months after OAGB | 37 ± 9.2 (12.9–53.2) | 34.1 ± 11.7 (14.1–61.1) | 0.119 |
| Additional %TWL 60 months | 30.6 ± 10.8 (− 4.3–52.5) | 30.4 ± 11.5 (7.9–52.1) | 0.768 |
| Postoperative leak | 9 (6%) | 3 (4.6%) | 0.685 |
| Conversion to RYGB for resistant reflux | 7 (4.7%) | 2 (3%) | 0.264 |

All patients underwent conversion to OAGB (data are presented as mean ± SD for continuous variables, as absolute number and percentages for categorical variables). Significant values are reported in bold

Table 9 Postoperative leak and conversion to RYGB for reflux resistant to medical treatment according to the timing of OAGB and reasons for delayed OAGB

|  | Postoperative leak, *n* (%) | Conversion to RYGB for resistant biliary reflux, *n* (%) |
| --- | --- | --- |
| Cause of conversion to OAGB | | |
| Proximal gastric dilatation (*n* = 116) | 6 (5.1) | 5 (4.3) |
| Other (*n* = 99) | 6 (6) | 4 (4) |
| Patients with delay > 12 months | | |
| Proximal gastric dilatation (*n* = 34) | 1 (2.9) | 1 (2.9) |
| Other (*n* = 24) | 0 (0) | 0 (0) |
| Patients with synchronous OAGB | | |
| Proximal gastric dilatation (*n* = 36) | 3 (8.3) | 1 (2.7) |
| Other (*n* = 33) | 1 (3) | 1 (3) |
| Patients with delayed OAGB | | |
| Proximal gastric dilatation (*n* = 46) | 2 (4.3) | 3 (6.5) |
| Other (*n* = 38) | 5 (13.1) | 3 (7.9) |
| Perforation (*n* = 5) | 1 (20) | 1 (20) |
| Other than dilatation and perforation | 4 (12.1) | 2 (6) |
| Patients with delayed OAGB | | |
| Preoperative proximal gastric dilatation (*n* = 38) | 1 (2.6) | 3 (7.9) |
| Other (*n* = 41) | 6 (14.6) | 3 (7.3) |
| Preoperative inflammation (*n* = 37) | 6 (16.7) | 2 (5.4) |
